# Supplementary material for: Glia Not Neurons: Uncovering Brain Dysmaturation in a Rat Model of Alzheimer’s Disease
Source: Biomedicines. 2021 Jul 15;9(7):823. doi: 10.3390/biomedicines9070823 (PMC8301397; doi:10.3390/biomedicines9070823)
Supplement: Supplementary file 1 [file biomedicines-09-00823-s001.zip › biomedicines-1274872-supplementary.pdf]

Supplementary Materials:

Table S1. Distributions of the density of cells from the neuronal lineage throughout the regions of the hippocampus and cortical layers of OXYS and Wistar pups (counts per 1 mm²).

Region	Wistar rats			OXYS rats		
	Neuroblasts	Immature neurons	Mature neurons	Neuroblasts	Immature neurons	Mature neurons
**PND0**						
Cortex	232±23	2122±84	1798±164	251±28	2465±216	1567±200
Layer I	552±062	629±41	251±37	618±97	715±95	226±28
Layers II-III	272±78	3278±220	1591±202	268±75	3324±330	1540±203
Layers V-VI	145±019	2176±260	2206±203	176±25	2582±199	1854±257
Hippocampus	280±34	1341±56	833±84	386±16*	1592±115	819±82
CA1 region	239±39	1203±72	862±74	370±21*	1526±118*	815±86
Pyramidal layer of CA1	167±22	2039±66	1565±175	228±37	2591±135*	1519±194
Molecular layer of CA1	288±56	636±99	396±60	451±38*	888±141	378±40
CA3 region	386±44	869±129	520±99	386±27	1073±124	574±94
Pyramidal layer of CA3	293±22	1443±89	1057±220	327±47	1910±218	1204±146
Molecular layer of CA3	449±68	548±111	234±38	446±63	665±109	253±64
DG	313±44	1992±179	985±123	435±24*	2299±122	1081±102
Granular layer of DG	199±39	2586±208	1430±262	268±30	2555±151	1464±187
Molecular layer of DG	483±60	695±90	422±55	692±29*	1275±217*	498±94
Hilus	276±61	2630±276	1104±198	415±45	3198±332	1173±214
**PND1**						
Cortex	288±26	1290±86	2107±135	300±49	1394±172	1886±178
Layer I	447±67	554±51	440±46	497±111	545±89	302±98
Layers II-III	330±30	2440±324	2074±231	403±79	2448±375	1690±352
Layers V-VI	241±28	1153±85	2499±138	214±32	1280±163	2399±203
Hippocampus	276±8	801±86	1140±92	290±30	794±36	1023±156
CA1 region	236±19	636±77	1019±78	236±30	691±79	954±177
Pyramidal layer of CA1	257±39	1088±81	1974±90	248±58	1159±137	1782±334
Molecular layer of CA1	226±11	379±83	471±74	232±20	416±62	497±113
CA3 region	380±69	673±99	877±82	279±49	508±105	798±123
Pyramidal layer of CA3	403±93	1105±142	1579±214	337±55	1000±229	1730±291
Molecular layer of CA3	361±96	376±78	385±68	250±54	273±50	354±84
DG	304±9	1307±174	1498±95	448±66	1369±104	1512±185
Granular layer of DG	253±16	1610±203	2159±155	431±84	1508±161	1920±263
Molecular layer of DG	348±35	701±152	582±45	485±43*	841±113	824±157
Hilus	340±39	1551±253	1532±225	449±93	1785±160	1670±216
**PND3**						
Cortex	349±69	733±139	820±112	407±24	977±173	1032±196
Layer I	435±61	352±64	193±68	365±71	327±58	141±47
Layers II-III	558±99	1430±335	808±102	656±59	1809±377	851±246

Layers V-VI	257±58	682±173	1107±182	339±29	898±162	1371±200
Hippocampus	335±23	492±54	594±111	355±21	502±66	416±72
CA1 region	280±14	448±49	548±110	310±24	455±61	400±60
Pyramidal layer of CA1	358±31	830±98	1156±199	427±30	858±133	841±150
Molecular layer of CA1	241±10	242±35	218±54	252±21	255±28	180±20
CA3 region	322±33	367±38	598±60	291±27	281±35	232±52*
Pyramidal layer of CA3	362±31	577±69	1193±132	492±98	564±75	476±124*
Molecular layer of CA3	292±42	206±22	147±22	208±28	131±26	74±10*
DG	456±48	662±102	683±168	529±43	779±133	559±147
Granular layer of DG	492±49	922±177	1108±291	660±55*	1102±190	983±280
Molecular layer of DG	313±53	212±29	209±53	351±31	347±71	193±75
Hilus	580±88	905±114	733±170	562±50	852±150	479±121
**PND5**						
Cortex	50±10	134±30	1943±72	59±15	118±28	1414±92*
Layer I	43±11	52±9	327±80	53±20	45±18	222±39
Layers II-III	73±18	285±59	3279±149	77±15	265±71	2608±225*
Layers V-VI	47±10	116±32	2241±136	58±18	113±32	1989±205
Hippocampus	68±17	93±15	1281±37	55±8	68±5	984±65*
CA1 region	48±13	56±13	1132±31	40±9	44±7	870±47*
Pyramidal layer of CA1	67±15	145±32	3209±117	55±11	109±23	2604±156*
Molecular layer of CA1	40±13	20±6	275±27	35±9	23±4	245±17
CA3 region	79±26	58±9	796±68	52±12	37±6	708±62
Pyramidal layer of CA3	70±12	74±12	1522±149	64±12	48±7	1615±143
Molecular layer of CA3	75±38	47±12	247±23	45±12	30±11	164±28*
DG	112±25	205±39	1942±127	81±9	132±9	1375±107*
Granular layer of DG	119±37	318±83	3623±395	91±17	266±28	2917±265
Molecular layer of DG	70±19	64±18	458±125	54±4	36±8	222±38
Hilus	180±38	299±55	2360±417	108±26	113±19*	1208±216*
**PND7**						
Cortex	118±14	228±22	1322±92	120±18	240±49	1367±91
Layer I	112±17	61±1	105±18	142±34	70±22	165±13*
Layers II-III	160±22	526±41	1901±203	131±21	553±93	2039±284
Layers V-VI	97±15	199±41	2239±369	100±14	185±23	2134±177
Hippocampus	152±22	249±47	1097±102	128±21	277±60	1021±49
CA1 region	126±18	145±19	963±118	115±23	196±43	821±50
Pyramidal layer of CA1	131±20	238±25	2343±217	155±40	432±108	2178±112
Molecular layer of CA1	102±11	95±16	236±18	106±17	92±15	225±10
CA3 region	144±24	103±21	766±67	103±13	130±27	632±57
Pyramidal layer of CA3	141±20	141±39	1285±101	124±26	188±38	1190±86
Molecular layer of CA3	131±27	75±15	234±42	103±15	81±17	167±29

DG	179±22	548±120	1809±82	207±37	524±110	1688±112
Granular layer of DG	155±32	803±194	3205±92	138±11	800±167	362±197
Molecular layer of DG	198±25	133±32	320±42	154±31	162±29	271±33
Hilus	228±18	677±112	1945±139	222±19	415±56	1424±128*

*$p < 0.05$ for differences between the strains

Table S2. Distributions of the density of astrocytes and its progenitors throughout the regions of the hippocampus and cortical layers of OXYS and Wistar pups (counts per 1 mm^2).

Region	Wistar rats		OXYS rats	
	**Astrocyte progenitors**	**Astrocytes**	**Astrocyte progenitors**	**Astrocytes**
		**PND0**		
Cortex	62±10	55±7	35±6*	30±6*
Layer I	536±91	307±92	151±35*	75±20*
Layers II-III	117±24	85±16	54±14*	72±18
Layers V-VI	62±10	55±7	35±6*	30±6*
Hippocampus	322±61	269±40	162±25*	165±23*
CA1 region	151±27	128±11	91±15	90±17
Pyramidal layer of CA1	95±16	108±15	80±18	100±28
Molecular layer of CA1	180±33	138±14	99±15*	86±14*
CA3 region	540±138	411±123	196±35*	248±41
Pyramidal layer of CA3	353±95	390±112	176±47	240±59
Molecular layer of CA3	625±181	432±165	208±34*	255±40
DG	451±104	409±60	287±51	249±42*
Granular layer of DG	319±83	364±67	208±51	241±44
Molecular layer of DG	740±190	541±103	453±108	230±41*
Hilus	314±59	369±75	270±88	333±94
		**PND1**		
Cortex	214±36	133±24	175±61	84±14
Layer I	561±88	305±67	424±154	180±44
Layers II-III	180±45	120±18	118±26	79±9
Layers V-VI	96±17	71±15	81±20	45±9
Hippocampus	637±70	382±36	685±152	399±64
CA1 region	358±65	188±16	428±84	247±54
Pyramidal layer of CA1	203±62	116±15	223±50	131±30
Molecular layer of CA1	438±68	225±18	539±107	313±69
CA3 region	1111±118	550±71	885±247	592±89
Pyramidal layer of CA3	523±104	301±87	52±206	300±155
Molecular layer of CA3	1468±167	701±82	150±269	770±105
DG	174±134	770±94	169±250	582±92
Granular layer of DG	977±176	707±106	908±269	462±100
Molecular layer of DG	1400±142	904±143	1409±251	723±101
Hilus	774±123	722±98	985±246	678±186
		**PND3**		

Cortex	128±28	101±22	159±10	145±17
Layer I	388±189	201±51	570±75	501±43*
Layers II-III	116±35	102±23	108±11	121±23
Layers V-VI	64±14	47±6	57±6	39±6
Hippocampus	449±103	307±44	433±42	533±62*
CA1 region	275±62	188±26	227±30	247±33
Pyramidal layer of CA1	122±32	94±18	78±14	49±6*
Molecular layer of CA1	344±74	233±30	296±41	337±50
CA3 region	902±192	635±129	694±60	901±101
Pyramidal layer of CA3	622±109	521±134	455±62	612±109
Molecular layer of CA3	126±227	692±131	832±71	165±127
DG	552±110	411±74	620±76	783±68*
Granular layer of DG	475±135	384±79	323±51	497±67
Molecular layer of DG	659±122	484±107	119±132	1244±133*
Hilus	483±69	335±48	549±77	658±63*
		**PND5**		
Cortex	113±14	124±15	90±12	145±28
Layer I	437±103	439±76	247±58	394±66
Layers II-III	105±23	110±17	65±15	97±22
Layers V-VI	36±4	46±7	47±9	40±5
Hippocampus	172±28	514±63	197±65	308±72*
CA1 region	119±26	356±61	141±40	245±74
Pyramidal layer of CA1	71±28	101±22	95±28	151±66
Molecular layer of CA1	139±26	462±82	158±45	278±78
CA3 region	273±43	814±96	236±79	336±64*
Pyramidal layer of CA3	90±21	222±36	92±38	149±76
Molecular layer of CA3	343±54	132±128	300±106	419±70*
DG	215±39	675±117	244±87	411±90
Granular layer of DG	150±41	464±122	179±77	190±67
Molecular layer of DG	284±53	968±161	228±65	649±156
Hilus	209±40	501±107	350±151	389±96
		**PND7**		
Cortex	251±36	310±57	167±36	113±13*
Layer I	383±58	485±91	297±65	230±56*
Layers II-III	228±53	221±32	133±45	97±23*
Layers V-VI	124±25	120±20	128±42	60±13*
Hippocampus	472±69	477±85	407±66	263±37*
CA1 region	371±58	410±92	312±73	224±37
Pyramidal layer of CA1	194±51	195±62	201±63	90±21
Molecular layer of CA1	443±69	498±110	351±78	274±43
CA3 region	549±115	631±87	592±67	339±45*
Pyramidal layer of CA3	248±58	358±84	229±52	119±23*
Molecular layer of CA3	731±167	798±100	823±90	483±61*

DG	674±123	564±95	469±87	304±54*
Granular layer of DG	463±93	375±81	300±86	182±26*
Molecular layer of DG	757±141	671±125	479±83	339±69*
Hilus	916±234	715±177	891±184	454±102

*$p < 0.05$ for differences between the strains

Table S3. Distributions of the density of microglial cells throughout the regions of the hippocampus and cortical layers of OXYS and Wistar pups (counts per 1 mm^2).

Region	Wistar rats			OXYS rats		
	Resting microglia	Activated microglia	Total microglia	Resting microglia	Activated microglia	Total microglia
			PND0			
Cortex	76±11	71±13	147±22	42±6*	69±6	111±6
Layer I	210±31	213±026	423±32	162±34	256±28	418±40
Layers II-III	28±25	48±42	76±67	12±6	20±10	33±12
Layers V-VI	51±17	31±3	82±18	20±5	33±7	54±8
Hippocampus	113±8	146±18	259±19	60±6*	92±13*	151±17*
CA1 region	116±10	104±13	220±18	55±11*	78±11	133±20*
Pyramidal layer of CA1	41±11	88±13	129±17	38±10	59±7	97±14
Molecular layer of CA1	158±13	114±15	272±21	69±14*	91±14	160±24*
CA3 region	65±10	219±42	284±40	43±7	132±38	175±35
Pyramidal layer of CA3	40±19	133±48	174±49	48±19	116±44	164±42
Molecular layer of CA3	73±9	246±42	319±39	43±7*	137±37	180±35*
DG	160±17	147±17	307±23	74±10*	76±10*	150±10*
Granular layer of DG	58±15	79±25	137±38	48±12	24±10	71±15
Molecular layer of DG	369±49	307±32	675±54	162±26*	202±30*	363±31*
Hilus	50±13	42±16	93±26	19±9	32±14	51±20
			PND1			
Cortex	118±15	68±5	186±19	100±12	64±7	164±16
Layer I	300±34	201±26	502±56	357±46	212±32	570±71
Layers II-III	64±21	12±9	76±28	20±5	2±2	22±5
Layers V-VI	62±10	34±5	95±13	37±9	36±6	73±12
Hippocampus	142±11	146±18	288±22	118±14	93±10*	211±17*
CA1 region	144±15	119±15	262±22	126±18	82±10	209±20
Pyramidal layer of CA1	42±7	96±16	138±20	29±8	66±5	95±8
Molecular layer of CA1	198±22	131±16	329±27	181±26	92±14	273±29
CA3 region	82±14	216±40	298±34	64±14	128±19	192±22*
Pyramidal layer of CA3	26±9	119±27	145±28	19±6	78±15	96±16
Molecular layer of CA3	108±19	264±57	372±54	83±18	149±22	232±25*
DG	181±20	148±20	329±29	151±16	77±9*	227±15*
Granular layer of DG	98±16	118±25	216±35	64±19	27±10*	91±22*
Molecular layer of DG	399±45	267±47	666±53	446±43	200±31	646±50

Hilus	72±17	70±21	142±32	35±11	48±18	83±26
**PND3**						
Cortex	136±17	43±4	179±16	92±10*	45±6	137±14
Layer I	311±41	87±11	398±41	281±40	81±22	362±43
Layers II-III	66±12	24±8	90±9	34±16	12±7	46±23
Layers V-VI	98±10	36±6	134±10	37±6*	41±6	78±10*
Hippocampus	183±10	154±19	337±28	131±11*	124±17	254±25*
CA1 region	187±13	160±23	347±35	137±15*	115±18	252±30
Pyramidal layer of CA1	70±13	168±36	238±42	27±11*	116±36	143±47
Molecular layer of CA1	243±18	155±19	397±33	184±18*	115±12	299±26*
CA3 region	98±11	211±30	310±34	73±9	188±14	261±20
Pyramidal layer of CA3	60±12	93±14	153±20	30±11	89±17	119±19
Molecular layer of CA3	114±13	266±45	380±48	90±9	227±22	317±28
DG	224±11	94±11	318±17	157±13*	94±18	251±25*
Granular layer of DG	66±21	44±12	110±28	38±8	29±8	67±13
Molecular layer of DG	500±34	163±22	662±40	403±30*	180±35	583±44
Hilus	72±13	59±16	131±13	25±6*	67±17	92±19
**PND5**						
Cortex	1.54±0.14	0.33±0.03	1.87±0.15	0.97±0.06*	0.41±0.05	1.38±0.05*
Layer I	3.74±0.59	0.66±0.17	4.40±0.65	1.54±0.17*	0.84±0.15	2.38±0.18*
Layers II-III	1.52±0.19	0.11±0.04	1.63±0.16	0.82±0.15*	0.13±0.04	0.95±0.18*
Layers V-VI	1.05±0.13	0.30±0.05	1.35±0.12	0.80±0.07	0.29±0.03	1.10±0.08
Hippocampus	148±8	128±9	275±9	109±11*	112±13	221±18*
CA1 region	137±7	144±8	281±9	125±10	109±12*	235±15*
Pyramidal layer of CA1	35±7	170±12	205±15	55±11	132±17	187±23
Molecular layer of CA1	180±11	133±9	313±13	152±14	101±11*	253±18*
CA3 region	90±7	143±16	234±16	65±12	136±25	202±26
Pyramidal layer of CA3	59±10	49±10	109±18	46±13	42±12	88±11
Molecular layer of CA3	105±12	189±26	294±26	74±12	181±40	255±43
DG	204±15	86±14	291±17	116±16*	102±13	218±21*
Granular layer of DG	63±17	38±10	101±25	57±8	49±11	106±16
Molecular layer of DG	414±35	143±23	557±23	218±39*	182±20	400±38*
Hilus	52±10	64±22	116±29	46±13	61±11	108±15
**PND7**						
Cortex	276±13	27±6	304±12	222±12*	33±7	254±10*
Layer I	323±35	39±7	362±35	227±22*	70±26	297±33
Layers II-III	257±15	35±11	292±21	245±14	26±7	271±13
Layers V-VI	288±26	13±5	301±24	203±17*	17±4	220±17*
Hippocampus	347±27	103±7	450±27	222±16*	87±6	309±19*
CA1 region	344±31	120±8	463±31	228±22*	091±8*	319±25*
Pyramidal layer of CA1	143±11	146±11	289±17	85±14*	110±13	195±17*
Molecular layer of CA1	415±43	111±9	526±45	276±26*	84±8*	360±30*

CA3 region	231±15	105±15	337±14	147±12*	97±10	244±17*
Pyramidal layer of CA3	144±24	49±16	193±27	95±20	79±13	168±32
Molecular layer of CA3	269±18	128±21	398±24	170±15*	108±10	278±17*
DG	422±35	63±6	485±37	254±25*	76±7	329±28*
Granular layer of DG	145±33	29±6	173±34	76±10	26±6	101±11
Molecular layer of DG	471±51	88±13	859±56	480±49*	140±21	620±62*
Hilus	191±24	64±13	255±32	129±13*	45±12	174±6*

*$p < 0.05$ for differences between the strains
